# Supplementary material for: A study protocol for a multi-specialty observational cohort comparing robotic stapler and bedside stapler outcomes in robotic-assisted surgeries
Source: PLoS One. 2025 Dec 23;20(12):e0339191. doi: 10.1371/journal.pone.0339191 (PMC12725535; doi:10.1371/journal.pone.0339191)
Supplement: S1 Table — (DOCX) [file pone.0339191.s001.docx]

**Supplementary Materials**

**S Table 1. Strings for Identification Staplers in Charged Descriptions**

| Stapler Modality | **Included** Strings | |
| --- | --- | --- |
| Robotic stapler | SUREFORM, SURFRM, SUREFOR, SUREFRM, SURE FORM, SRFRM, SUREFOAM, SF, 480445, 48345G, 480545, 48345T, 480460, 48360W, 48345M, 48360B, 48345W, 48360G, 48345B, 48360T |  |
| Bedside stapler | SIGNIA, IDRIVE, REINFORCED, REINF, POWERED, POWER, PWRD, PRWD, PWR, PWD, SIGPSHELL, PCEE60A, GST60T, SIGPHANDLE, PSEE60A, GST45W, SIGADAPTSTND, PLEE60A, GST45B, SIGADAPTXL, PVE35A, GST45D, SIGADAPTSHORT, GST60W, GST45G, PCEE45A, GST60B, GST45T, PSEE45A, GST60D, PLEE45A, GST60G, SIGC60VM, SIG60CTAVM, SIGC60MT, SIG60AXT, SIG60AMT, SIG60CTAMT, SIG45CTAV, SIG30CTAV, SIG30AV, SIGC45VM, SIG45CTAVM, SIG30CTAVM, SIG30AVM, SIGC45MT, SIG45AXT, SIG45CTAMT, SIG30AMT, SIG45AXT, SIGTRS45AMT, SIGTRS45AXT, SIGTRS60AMT, SIGTRS60AXT, COVIDIEN, ECHELON, ECHELN, ECHLN, ECHEL, ECHL, ECH, ENDO GIA, TRI, ENDOGIA, TRI-STAPLE, UNIVERSAL, TRISTAPLE, UNIVL, TRISTA, UNIV, TRISTAPL, ENDOPATH, TRIST, ENDOPTH, TRI TAPLER, ENDOPAT, TRI-STAPLES, ENDPTH, ROTIC, FLEX, ROTICULATOR, FLX, EGIAUSTND, ATG45, NAB45, EGIAUXL, EGIAUSHORT, ATS45, NAG45, GIAUSHORT, ATS45NK, NAW45, EC45A, TW35, NK45B, EC45AL, ATW45, NK45G, C45A, CTS45, NSB45, EC60A, CTS45NK, NSG45, SC60A, ET45B, NSW45, LONG60A, ET45G, SCB45, 6CB45, ETS45, SCG45, 6SB45, EZ45B, TSB35, 6TB45, EZ45G, TSG45, ATB35, LONG45A, TSW35, ATB45, LTS60A, TSW45, SIG60AXT, EGIA60AXT, TR45B, ECR60B, EGIA, EGIA45CTAVM, ECR45W, TR45W, ECR60D, EGIA60AVM, EGIA60CTAVM, ECR45B, 6R45B, ECR60G, EGIA60AMT, EGIA60CTAMT, ECR45D, ZR45G, ECR60M, EGIA45AVM,  030403, 030414, 030415, 030422, 030423, 030425, 030426, 030449, 030450, 030453, 030454, 030455, 030455, 030456, 030458, 040458, ECR45G, ZR45B, ECR60T, EGIA45AMT, ECR45M, 6R45M, EGIA45AXT, TR45G, ECR60W, EGIA30AMT, EGIA30AVM, EGIA30CTAVM, EGIA45CTAMT, EGIATRS45AMT, EGIATRS45AXT, EGIATRS60AMT, EGIATRS60AXT, EGIAUNIVXL | |

| Stapler Type | **Excluded** Strings | |
| --- | --- | --- |
| Circular stapler | CEEA, EEA, CIRCULAR, CIRC, CDH, ECS |  |
| Vascular stapler | VASCULAR, VASC |  |
