# Supplementary material for: A study protocol for a multi-specialty observational cohort comparing robotic stapler and bedside stapler outcomes in robotic-assisted surgeries
Source: PLoS One. 2025 Dec 23;20(12):e0339191. doi: 10.1371/journal.pone.0339191 (PMC12725535; doi:10.1371/journal.pone.0339191)
Supplement: Attachment — Submitted filename: Response to Editorial Request.docx [file pone.0339191.s002.docx]

Dear PLOS ONE Editorial Office,

Thank you for your feedback on our manuscript [Manuscript ID: PONE-D-25-28738], “A Study Protocol for a Multi-Specialty Observational Cohort Comparing Robotic Stapler and Bedside Stapler Outcomes in Robotic-Assisted Surgeries.”

We have revised the manuscript in accordance with your comments and resubmitted all required documents, including:

- An updated Methods section with study status and timelines
- The IRB exemption determination letter (uploaded under file type “Other”)
- A completed Human Participants Research Checklist (uploaded under file type “Other”)
- A point-by-point response to your editorial requests (included below)

Please let us know if any additional revisions or documents are needed. We look forward to your further evaluation.

Sincerely,
Peng-Lin Lin
*HEOR Data Science Manager, APAC
Intuitive Surgical*
Email: [lin.penglin@outlook.com](mailto:lin.penglin@outlook.com) / [penglin.lin@intusurg.com](mailto:penglin.lin@intusurg.com)

Point-by-Point Response to Editorial Requests

1. Study Status and Timeline (Methods Section)

We have updated the *Methods* section of the manuscript to explicitly state the timeline of the study, including planned data extraction periods and expected result availability for each procedure group. None of the results have been generated yet. All data collection is scheduled for the future. The added details include:

- Lung and colorectal resections: Data extraction starting September 1, 2025, ending October 31, 2025; results by December 31, 2025.
- Bariatric surgeries and gastrectomy: Data extraction starting January 1, 2026, ending February 28, 2026; results by April 30, 2026.

These timelines are now reflected in the second paragraph of the *Study Design* subsection under *Methods and Analysis* (Line 114-121).

2. IRB Approval Letter or Waiver

We have uploaded the IRB exemption determination letter from WCG IRB as an “Other” file. This document confirms that our study, which involves secondary analysis of de-identified data from the PINC AI Healthcare Database, qualifies for exemption from IRB review.

3. Human Participants Research Checklist

We have completed the PLOS ONE Human Participants Research Checklist and uploaded it as an “Other” file with our resubmission. It addresses all relevant questions pertaining to our retrospective study using de-identified data.
